# Branching out: the diverse roles of stem and progenitor cells in mammary gland development

**DOI:** 10.1007/s10911-026-09595-x

**Published:** 2026-03-11

**Authors:** Andrew Olander, Shaheen S. Sikandar

**Affiliations:** 1 Department of Molecular, Cell and Developmental Biology, University of California, Santa Cruz, USA; 2 Genomics Institute, University of California, Santa Cruz, USA; 3 Institute for the Biology of Stem Cells, University of California, Santa Cruz, USA

**Keywords:** Mammary stem cells, Progenitors, Differentiation, Heterogeneity, Lineage commitment

## Abstract

Tissue specific stem cells are critical in maintaining organ function throughout life. Understanding the dynamics and heterogeneity of stem cells and progenitors is essential to understanding how diseased cell states such as cancer evolve. Studies in the mammary gland have revealed significant heterogeneity in stem cell identity during embryonic development, puberty, adulthood and aging. In this review, we discuss the dynamics of lineage commitment in the mammary gland and the current understanding of mammary stem cells versus lineage committed progenitors at different stages. As breast cancer risk increases with age, we also review recent studies on the aged mammary gland in relation to lineage identity and provide perspectives for future research in understanding the niche of mammary epithelial cells.

## Introduction

The mammary gland is a secretory organ with the primary function of producing milk to provide nutrients to offspring. Unlike most organs, the mammary gland ductal tree fully develops during puberty in response to hormones and undergoes significant expansion during pregnancy. Mammary stem cells (MaSCs) and progenitors play a central role in mammary gland development and homeostasis to maintain two main epithelial cell types: basal and luminal cells. Their activity drives key developmental stages, including ductal tree formation during puberty, cyclic remodeling during estrus, and alveologenesis and involution during reproduction. Together, basal and luminal cells make up a bilayered network of epithelial arborizations, with basal cells enclosing a layer of luminal cells. The spatial architecture of this bilayered structure allows basal cells with mesenchymal morphology to contract around luminal cells as they produce breast milk, emptying their nutrients into the lumen for transport to the nipple.

MaSCs and progenitors are of great interest to the medical community, as they represent the putative cell-of-origin for breast cancer [1–4]. A comprehensive understanding of their function in the healthy mammary gland is therefore critical to address and target their tumorigenic potential when they become dysfunctional. Recent advances in the identification and characterization of MaSCs and progenitors have aided tremendously in this effort, from new technologies that reveal cellular heterogeneity to innovative mouse models that allow functional investigation of stem cell dynamics in vivo.

This review summarizes the recent advances in unmasking the heterogeneity of MaSCs, their progenitors, and lineage commitment during the different stages of mammary gland development. We summarize the different markers used to identify specific populations within basal and luminal lineages. Finally, we discuss recent advances in understanding how aging impacts mammary epithelial cell identity and differentiation in the mammary gland.

### Stem and progenitor cells during mammary gland development

#### Embryonic

Mammary gland development begins at embryonic day 10.5 (E10.5) in mice with the establishment of bilateral milk lines [5, 6]. Ectodermal cells organize into five structures known as placodes along each milk line by E11.5. By E13.5, the placodes have progressed into bud-like epithelial structures that, in concert with the surrounding mammary mesenchyme, begin to invade the underlying dermal mesenchyme [7]. As the mammary bud migrates downward into the dermal mesenchyme, the surrounding mammary mesenchyme plays a critical role in the specification of mammary epithelial cells through secreted factors. The mammary bud extends into the fat pad to form a minimally arborized structure with a hollow lumen [8]. This final step of embryonic development of the mammary gland begins at ~ E15.5 and proceeds until birth.

Analysis of stem cells in the embryonic state has been challenging due to the limited number of cells. However, recent advances in single-cell technologies and lineage tracing have provided critical insights into the identity of stem cells. Earlier studies in the embryonic mammary gland have revealed that MaSCs are bipotent, giving rise to both luminal and basal lineages in the mammary gland [1, 9–11]. More recent studies have dissected the complexity of lineage commitment at different stages during embryonic development. Using lineage tracing with stable barcodes injected at E9.5, a recent study suggests that the mouse mammary gland may be derived from about ~ 120 early progenitor cells that expand relatively uniformly, each with similar growth potential [12]. However, lineage-specific Cre promoters (*Krt5, Krt14, Acta2, Notch1*) combined with confetti reporters initiated at E12.5 revealed substantial heterogeneity in clonal outgrowth and lineage specification, arguing against equipotency and instead pointing to the onset of early lineage segregation. Indeed, some of these uncommitted cells at E9.5 can become lineage restricted as early as E12.5 [13] and the results could reflect genuine biological differences in developmental stages [13, 14]. Studies at later time points (E15.5 and E18.5) demonstrate that while MECs can transcriptionally remain in an undifferentiated hybrid cell state with luminal and basal gene signatures [13–15], lineage tracing of MECs showed that multipotency in vivo is negligible at E15.5 [13]. In contrast, a single-cell RNA sequencing study from Pal et al*.* comparing multiple timepoints throughout development (from embryonic to adult) has argued that the embryonic mammary epithelium, even at E18.5, comprises only basal-like MECs with epigenetic features linked to multilineage differentiation potential [16, 17]. Interestingly, chromatin analysis of MECs at E18.5 demonstrated that the cells have both basal-like and luminal-like priming suggesting that the cells may appear to be hybrid, but they demonstrate partial lineage commitment [15].

Several studies have explored the transcriptional programs that drive lineage commitment during embryonic development. Gain-of-function NOTCH1 models in the embryonic mammary gland [13] have shown that intrinsic Notch activation governs luminal fate determination. On the other hand, inducing expression of ΔNP63 promotes a basal cell fate in both hybrid embryonic mammary cells and committed luminal cells [14], suggesting distinct transcriptional programs are driving cell fate decisions during early development. Similarly, lineage tracing studies using *Blimp1* have shown that lineage commitment to luminal cells occurs at E17.5 and these cells are capable of long-term contribution to the adult mammary gland [18]. To further understand when lineage commitment occurs during embryonic development, a recent study performed single-cell RNA-sequencing on embryonic MECs at multiple timepoints. They found that lineage commitment during embryonic development is progressive and, in contrast to the Pal et al*.* study, were already able to identify three transcriptionally discrete cell populations at E15.5—basal-like, luminal-like and hybrid cells [19]. They also found that these basal-like and luminal-like cells are organized in spatially distinct regions similar to their positions in the post-natal mammary gland, supporting the model that lineage specification occurs during embryonic development. It would be interesting to collate single-cell RNA-sequencing data from all these studies using the earliest timepoints to the adult state in a single dataset to determine the differences and similarities in lineage-committed MECs in the embryo and in the adult.

#### Puberty

In response to alterations in the relative proportions of hormones and growth factors, such as beta-estradiol, progesterone, and insulin-like growth factor, the mammary gland enters the pubertal stage of development [20–25]. This stage is characterized by the elongation of the mammary epithelium from its rudimentary branches to occupy the entire fat pad.

The growth required during puberty is carried out by highly proliferative structures called terminal end buds (TEBs) that are composed of cap cells and body cells. Cap cells form a monolayer on the outer-most layer of the club-like structure and give rise to differentiated basal cells as the TEB progresses through the mammary fat pad [26]. The cap cell population was initially proposed to represent a MaSC-enriched population, as transplantation experiments have demonstrated their enhanced potential for mammary gland reconstitution compared to ductal basal cells [27]. However, lineage tracing studies on cap cell populations have contradicted this notion and have shown that cap cells marked by s-SHIP and P63 are lineage-restricted to basal cell fates [28]. While cap cells have been observed to migrate within the body cell cluster, these cells undergo apoptosis and do not contribute to the luminal lineage like typical body cells [28–30].

**Fig. 1:**
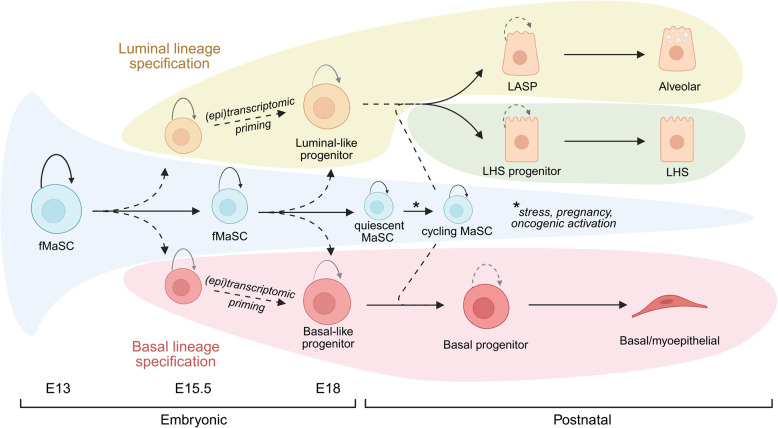
Mammary epithelial cell differentiation hierarchy in early development. Multipotent fetal mammary stem cells (fMaSCs) give rise to basal-primed and luminal-primed progenitors during the early stages of lineage specification (around E15.5-E17.5). Basal, luminal-hormone sensing (LHS), and luminal-adaptive secretory precursor (LASP) lineages are independently maintained by lineage-restricted progenitors in the post-natal mammary gland. Rare populations of quiescent mammary stem cells (MaSCs) persist from fetal MaSCs (fMaSC) and have the capacity to become cycling MaSCs, contributing to both basal and luminal lineages (see Table 1). Dashed arrows represent differentiation dynamics that are active areas of research

Beneath the monolayer of cap cells reside multiple layers of body cells. Body cells give rise to the luminal adaptive secretory precursor (LASP, also referred to as HR-low, alveolar/secretory (Fig. 1); see Table 1) and luminal hormone-sensing (LHS, also referred to as HR-high) populations. Similar to the luminal populations they give rise to, body cells express typical luminal keratin markers such as *Krt8, Krt18*. Despite extensive studies, including the generation of multiple single-cell RNA/ATAC sequencing datasets, little has been reported in terms of heterogeneous sub-populations within TEBs. Nevertheless, some of these efforts have revealed new insights into the distinctions between epithelial cells from ductal structures and those from TEBs. For example, cap cells displayed decreased expression of contractility-related genes compared to ductal basal cells and possessed a unique chromatin accessibility landscape around luminal lineage-specific loci [16]. Moreover, basal and luminal cells from TEBs displayed lower gene signature scores for their respective lineages compared to the same cell types from ductal structures, suggesting that the cells are in an immature/progenitor cell state. Previous studies have identified sSHIP + cap cells in the TEB that are enriched in stem cell activity by transplantation assays [27]. The sSHIP + cells are also enriched in expression of the Par polarity protein, PAR3L and loss of PAR3L significantly impacts regeneration in transplantation assays [56]. However, whether *Par3L* + cells contribute to both basal and luminal lineages during development in vivo is unknown. Similarly, Bu et al*.* identified a population of KRT6A + luminal cells present in the TEB, that have reconstitution capacity in transplantation assays [57] but contribution of KRT6A + cells in ductal elongation is unknown. Importantly, unbiased lineage tracing studies combined with single-cell analysis during puberty have suggested terminal end bud cells function as highly proliferative, lineage-committed MaSCs [58]. These cells are heterogeneous in their expression profile precluding the use of a single marker to label MaSCs [58]. Single-cell RNA-sequencing studies in the TEB [16] and preliminary work from our lab [59] has shown that unique populations of cells are enriched within the body of TEBs but not in the adult. However, the functional contribution of these populations to pubertal development remains to be determined.

**Table 1:** Molecular identifiers of mammary epithelial lineages and sub-lineages across development

Main lineage (alternative nomenclature)	Sub-lineage	Markers		Developmental stage	Publication
		**Flow cytometry**	**Gene signature**		
Basal (myoepithelial, basal/stem)	General basal	Lin-CD49f^hi^EpCAM^med^ Lin-CD49f^hi^CD24^+/med^ Lin-CD24^+/med^CD29^hi^	*Acta2, Trp63, Krt5, Krt14, Krt15, Krt17, Sparc, Vim, Oxtr, Mylk, Myl9, Postn, Cxcl14, Igfbp3, Ngfr, Axl, Bdnf, Bmp7, Cxcl12, Dkk1, Dll1, Fgf1, Efna5, Epha4, Fzd1, Fzd2, Fzd7, Fzd8, Gpc3, Hgf, Id4, Jag1, Id4, Jag1, Jag2, Wnt6, Wnt10a, Pdgfrb, Snai2*	~ E15.5 through adulthood	Asselin-Labat et al. 2007 [31] Kendrick et al. 2008 [32] Bach et al. 2017 [33] Giraddi et al. 2018 [15] Li et al. 2020 [34] Carabaña et al. 2024 [19] Gray et al. 2023 [35] Song et al. 2023 [36]
	General MaSC	Lin-CD49f^hi^EpCAM^hi^ Lin-CD49f^hi^EpCAM^hi^/cKIT^+^ Lin-CD49f^hi^EpCAM^hi/^CD61^+^	*Cd44, Nt5e, Zeb1, Zeb2, Itga6, Itgb1, Krt14, Krt5, Trp63, Sox9, Snai2, Lgr5*	Postnatal onward	Stingl et al. 2006 [37] Shackleton et al. 2006 [38] Spike et al. 2012 [1] Gray et al. 2023 [35] Guo et al. 2012 [39] de Visser et al. 2012 [40]
	Quiescent MaSC	Lin-CD24^+^CD29^hi^Tspan8^hi^	*Lgr5, Tspan8, Sfrp1, Sfrp2, Sfrp5, Sfrp4, Cpe, Ccdc88c, Sox17, Dkk2, Hhex, Cpz, Wnt2, Serping1, Gpx3, Slc10a6, Tgtp1, Rgs5, Dpt, Tnxb, Lama2, Atp2a3, Cfh, Tek, Gbp4, Vtn, Scara5, Htra3, Cbfa2t3, Bmp6, Lmo2, Bmp4, Hba−a1, Gli2, Kcnj8, Hsd11b1*	Adult	Fu et al. 2017 [11]
	Bipotent MaSC	Lin-CD49f^hi^EpCAM^med^CD34^−^CD200^+^	*Bcl11b, Cd200*	~ E10.5 onward; nipple region of the adult mammary gland; expanded by pregnancy	Lin et al. 2025 [41]
		mCherry + in *Dll1-mCherry *transgenic mice	*Dll1*	Adult, expanded during pregnancy	Chakrabarti et al. 2018 [42]
	Slow cycling MaSC	Lin-CD24^+^CD29^hi^CD1d^+^	*Cd1d, Nt5e, Cd22, Cd93, Cd59a*	Pubertal, adult	dos Santos et al. 2013 [43]
	Cycling MaSC	Lin-CD24^+^CD29^hi^Procr^+^ Lin-CD24^+^CD29^hi^PD-L1^+^	*Procr, Igfbp7, Notch3, Mmp9, Mmp3, Foxc2, Zeb1, Zeb2, Twist1, Igfbp4, Vim, Fn1, Gng11, Col1a2, Col3a1, Cdh2, Serpine1, Pdl1, Zeb1, Zeb2, Foxc2, Gng11, Igfbp4, Vim, Mmp9*	Sparse at E18.5 and P1.5. Largely present at postnatal (peak at pre-pubertal stages and within ducts)	Wang et al. 2021 [44] Wang et al. 2015 [45] Bach et al. 2017 [33] Pal et al. 2021 [16]
	Embryonic multipotent progenitor (EMP)	Lin-CD49f^hi^/Lgr5-GFP^hi^	*Lgr5, Ep300, Lef1, Sox11, Uhrf1, Flrt1, Ctxn1, Notch1*	~E14-E17	Rodilla et al. 2015 [46] Wuidart et al. 2018 [14]
	Fetal MaSC (fMaSCs)	Lin-CD24^hi^EpCAM^hi^CD49f^hi^	*Eya2, Itga6, Ngr1, Sostdc1, Sox10, Myb, Lsr, Sfrp1, Bcl11a, Pthlh, Sema3b, Slitrk2*	E16-P1	Spike et al. 2012 [1] Makarem et al. 2013 [47] Giraddi et al. 2018 [15] Carabaña et al. 2024 [19]
	Unipotent progenitor	Lin-CD49f^hi^EpCAM^med^CD34^+^CD200^−^Sema3a +	*Cd34, Sema3a*	Pubertal TEBs, alveologenesis	Lin et al. 2025 [41]
		Lin-CD49f^hi^EpCAM^med^CD36^hi^/MTR^hi^	*Cd36*	8–12-week-old adults	Waas et al. 2024 [48]
Luminal adaptive secretory precursor, LASP (Hormone-receptor-low, HR-low, alveolar, secretory luminal, luminal progenitor)	General luminal	Lin-CD49f^lo/med^EpCAM^hi^ Lin-CD24^+^CD29^lo^	*Epcam, Krt8, Krt18, Krt19, Cdh1*	Emerge as early as E12.5, but lineage commitment largely occurs E15.5 - E17.5	Wang et al. 2015 [45] Stingl et al. 2006 [37] Shackleton et al. 2006 [38] Bach et al. 2017 [33] Li et al. 2020 [34] Lilja et al. 2018 [13]
	LASP progenitor	CD14^+^ in combination with cKIT^+^/CD49b^+^/CD61^+^ cKIT^+^/Sca-1^−^	*Elf5, Aldh1a3, Rspo1, Cd14, Itgb3, Ceacam1, Cd200, Egfr, Notch1*	Postnatal-adult, though initially transcriptionally undifferentiated	Asselin-Labat et al. 2011 [49] Regan et al. 2012 [50] Shehata et al. 2012 [51] Rodilla et al. 2015 [46] Bach et al. 2017 [33] Van Keymeulen et al. 2017 [52] Giraddi et al. 2018 [15] Li et al. 2020 [34] Gray et al. 2023 [35]
		CD55^+^/CD14^+^	*-*	Pre-pubertal onward	Pal et al. 2017 [53]
	Differentiated LASP	CD14^−^/cKIT^−^/CD49b^−^/CD61^−^	*Fabp3, Thrsp, Wap, Glycam1, Olah, Wfdc18, Mfge8, Lalba, Lif, Csn1s1, Csn1s2a, Csn3, Csnb, Wnt7b, Erbb3, Irak2, Ly96, Tlr4, Tnf, Ceacam1*	Pubertal onward	Sleeman et al. 2007 [54] Kendrick et al. 2008 [32] Asselin-Labat et al. 2011 [49] Bach et al. 2017 [33] Li et al. 2020 [34] Song et al. 2023 [36]
Luminal hormone-sensing, LHS (Hormone-receptor-high, HR-high, mature luminal, luminal hormone-responsive)	General luminal	Lin-CD49f^lo/med^EpCAM^hi^ Lin-CD24^+^CD29^lo^	*Epcam, Krt8, Krt18, Krt19, Cdh1*	Emerge as early as E12.5, but lineage commitment largely occurs E15.5 - E17.5	Wang et al. 2015 [45] Stingl et al. 2006 [37] Shackleton et al. 2006 [38] Bach et al. 2017 [33] Li et al. 2020 [34] Lilja et al. 2018 [13]
	LHS progenitor	CD133^+^/Sca1^+^in combination with cKIT^+^/CD49b^+^/CD61^+^	*Areg, Esr1, Pgr, Prlr, Foxa1, Wnt4, Cited1, Ly6a, Aldh1a3, Prom1, Itgb3, Sox9, Gata3, Notch3*	Postnatal-adult, though initially transcriptionally undifferentiated	Regan et al. 2012 [50] Shehata et al. 2012 [51] Lafkas et al. 2013 [55] Bach et al. 2017 [33] Van Keymeulen et al. 2017 [52] Giraddi et al. 2018 [15] Gray et al. 2023 [35]
	Differentiated LHS	CD133^+^/Sca-1^+^/cKIT^−^/CD49b^−^/CD61^−^ Sca-1^+^/cKIT^−^/CD49b^−^	*Areg, Esr1, Pgr, Prlr, Foxa1, Wnt4, Cited1, Ly6a, Prom1, Cldn10, Gata3, Cd24a, Erbb3, Notch3, Wnt5, Wnt7b, Myb, Krt7*	Pubertal onward	Asselin-Labat et al. 2007 [31] Sleeman et al. 2007 [54] Kendrick et al. 2008 [32] Regan et al. 2012 [50] Shehata et al. 2012 [51] Bach et al. 2017 [33] Giraddi et al. 2018 [15] Pal et al. 2021 [16] Li et al. 2020 [34] Gray et al. 2023 [35] Song et al. 2023 [36]

#### Adult

The adult mammary gland undergoes cyclical changes in response to relative hormone levels, namely estrogen and progesterone, that coincide with estrous cycle phases. Historically, studies have pointed to elevated levels of progesterone during diestrus as the driver behind widespread proliferation, particularly within the luminal lineage, which was subsequently followed by a phase of apoptosis and epithelial regression [60].

Morphological analysis of mouse mammary glands at different stages of estrous revealed distinct patterns in epithelial architecture at the diestrus phase [60, 61], notably pronounced tertiary branching and alveolar-like structures. Estrous-dependent changes in epithelial architecture were also investigated using intravital microscopy over a 3-month (12 cycles) period, which revealed cyclic expansion and regression of local side branching with little changes to the organization of main ducts [62]. Moreover, these morphological changes correlate with increased progesterone levels and proliferation rates. Joshi et al*.* investigated this phenotype to discover a progesterone-dependent expansion of the stem-cell-enriched CD49f^high^ population with enhanced reconstitution abilities during diestrus [61]. This process is likely mediated in part by paracrine signaling programs from RANKL + luminal cells. Another group also reported a diestrus-specific expansion of epithelial populations [63], but that most cell divisions occurred within the ER + luminal lineage. Interestingly, the resulting daughter cells from these diestrus-specific cell divisions did not persist post-regression (following diestrus). Adding complexity to these findings, Beleut et al*.* discovered that progesterone-mediated proliferation occurs in two distinct phases: the first occurs in a Cyclin D-dependent fashion in PR + luminal cells and is followed by the second, more dramatic, wave of proliferation in PR- luminal cells via RANKL signaling [64]. Despite the consistent reports of estrous-dependent epithelial expansion and regression, the proliferation phase that occurs during diestrus does not occur during every cycle [65].

However, there are conflicting reports as to whether epithelial sub-populations are significantly altered, both at the population level as well as the cellular level. Recent work by the van Amerongen group challenges the notion that the epithelial populations are significantly altered during different stages of the estrous cycle. Flow cytometry analysis of luminal (lin −/EpCAM^high^/CD49f^med^), basal (lin −/EpCAM^med^/CD49f^high^) and non-adipose stromal (lin-/EpCAM −/CD49f −) cells across puberty, proestrus, estrus, metestrus, and diestrus revealed no major differences in overall proportions of total cell count [66]. Differential gene expression analysis of luminal and basal populations across estrous phases revealed a mere 21 and 255 differentially expressed genes, respectively, in stark contrast to both fibroblasts and adipocytes, which totaled well over 1000 differentially expressed genes each [66].

#### Pregnancy, lactation and involution

Throughout reproductive stages, the mammary gland undergoes dramatic tissue remodeling phases that require highly orchestrated periods of proliferation and apoptosis. The observation that specific populations of MECs survive these rounds of proliferation and cell death suggests that this process is carried out by long-lived progenitors [46, 67–70].

Lineage-tracing studies have established the existence of *Blimp1* + */Notch1* + unipotent progenitors that contribute to the expansion of the alveolar luminal population during alveologenesis [18, 46]. These long-lived progenitors emerge during embryonic development, contribute to the ELF5 +/ER-/PR- luminal lineage, and persist after multiple rounds of pregnancy. When transplanted, the *Notch1*-expressing luminal cells contribute to both luminal and basal lineages and display high regenerative capacity [46]. In contrast to *Blimp1* + */Notch1* + progenitors, a separate population of alveolar precursors known as pregnancy-induced mammary epithelial cells (PI-MECs), accumulate during later stages of pregnancy to drive lactogenesis, but are absent in nulliparous mice [71, 72]. PI-MECs, which can be traced using the *Wap-Cre* mouse model, survive mammary gland regression during involution and contribute to alveolar development during subsequent pregnancies. While pregnancy-related morphological changes are most evident in the luminal lineage, rare populations of basal progenitors, such as those expressing *Nfatc1* or *Lgr6*, have been implicated in alveolar development [73, 74]. Despite their minimal contribution under homeostatic conditions, *Nfatc1*- and *Lgr6-*expressing basal cells clonally expand during pregnancy, and their contribution to alveoli is increased in subsequent pregnancies. However, in contrast to *Nfatc1*, *Lgr6* is also expressed by a rare population of lineage-restricted luminal progenitors [73]. *Lgr6* + luminal progenitors display similar clonal dynamics during pregnancy and in response to 17β-estradiol (E) and progesterone (P) exposure compared to their basal counterparts. Clonal analysis of *Lgr6*-traced MECs in pregnant mammary glands revealed single-lineage clusters composed of basal or luminal cells, but not both, affirming the existence of two distinct *Lgr6* + populations of lineage-restricted progenitors [73].

In addition to pregnancy-specific alterations in unipotent progenitor populations, there have also been multiple reports of bipotent progenitors that display distinct differentiation patterns during reproductive stages. A lineage-tracing study that tracked the fate of *Axin2* + MECs throughout multiple rounds of pregnancy discovered that AXIN2 marks Wnt/β-catenin responsive basal cells that contribute to alveologenesis. As basal and luminal cells post-pregnancy were found adjacent to each other and *Axin2* + cells persist over multiple rounds of pregnancy the authors concluded that AXIN2 marks a long-lived stem cell population in the mammary gland with bipotent capacity under certain contexts [75]. Similarly, clonal analysis using *Krt5, Krt14,* and *Lgr5*-driven confetti reporters suggests that bipotent basal stem cells not only exist in the adult mammary gland, but contribute to the luminal lineage during alveologenesis [76]. Interestingly, bipotency appears to not be limited to a subset of basal stem cells but also can be observed in the luminal lineage. Using a *Krt8-CreER*^*T2*^*;Rosa26-Rainbow* reporter mouse model, Song et al*.* identified luminal-derived basal cells (LdBCs) that become activated in response to pregnancy-related hormones [77]. The authors found that during pregnancy a small percentage of basal cells are derived from luminal cells labelled with *Krt8-CreER*^*T2*^. Moreover, these LdBCs had in vivo reconstitution capacity like basal cells and displayed a transcriptomic profile of both basal and luminal cells (hybrid). Their functional data is in contrast to several other studies that have previously demonstrated that in the post-natal mammary gland, luminal cells are unipotent. It is important to note that the *Krt8-CreER*^*T2*^ lineage tracing mouse used in Song et al*.* study is a BAC transgenic line and distinct from the previously used *Krt8-CreER*^*T2*^ [52] to lineage trace luminal cells. Further studies with more specific markers and/or unbiased strategies are needed to confirm whether luminal cells indeed have the capacity to generate basal cells during pregnancy.

In contrast to the long-lived progenitor populations, there appear to be short-lived progenitor populations that emerge during discrete periods of reproductive remodeling stages. For example, s-SHIP + MECs, which are typically absent in the post-pubertal adult mammary gland, re-emerge during early stages of pregnancy [27]. This population persisted until pregnancy day 15.5, contributing mainly to the outer layer of alveolar bud tips that are αSMA +. Similarly, single-cell RNA-seq profiling of mammary glands throughout reproduction have supported the existence of cell populations that are uniquely enriched during pregnancy, lactation, or involution. For example, a population of “alveolar lineage primed” basal cells emerges during lactation but are absent by involution [16]. Moreover, the authors identify an alveolar intermediate population that emerges during late pregnancy and expands during lactation.

Involution in the mammary gland is characterized by an inflammatory signature [78–80] accompanied by substantial changes in immune cell composition [81, 82] and extensive extracellular matrix remodeling [81]. This period represents a transient “reprogramming window” characterized by a wound-healing program that can create a permissive environment for tumor progression and metastasis [83–85]. Notably, abrupt involution has been shown to exacerbate inflammation and estrogenic signaling, resulting in an increased frequency of luminal progenitor cells (CD24 +/CD29^low^/CD61 +) as compared to gradually involuted glands [86]. Lineage tracing studies using adipocyte-binding protein *P2-Cre* [87] and *Pdgfrα-CreER*^*T2*^ [88] have further revealed that adipocyte progenitors can transition into epithelial cell states during pregnancy. However, because adipocyte/mesenchymal lineage-specific genes may be expressed at low levels in rare epithelial subpopulations, additional studies are required to definitively establish whether true transdifferentiation occurs within this reprogramming window.

Another study explored whether post-pregnancy MECs retain an enhanced response to pregnancy hormones due to intrinsic or microenvironmental factors [89]. To test this, pre- or post-pregnancy CD1d⁺ MaSCs were transplanted into cleared fat pads of virgin mice, which were then exposed to pregnancy hormones. Both groups showed similar lineage composition after engraftment, but glands from post-pregnancy MaSCs exhibited a 1.4-fold increase in ductal structures, indicating a heightened responsiveness that persisted outside their native environment. Interestingly, post-pregnancy/involution MECs in absence of pregnancy associated hormones have an overall decreased capacity of regeneration in transplantation assays and their depletion could be important in pregnancy induced breast cancer protection [90].

The extensive heterogeneity in distinct populations of stem and progenitors throughout reproductive stages underscores the dramatic complexity of the mammary epithelial hierarchy and its regulation. These findings emphasize that stem and progenitor activity is highly dynamic and shaped by reproductive context.

#### Aging

Given the hypothesis that stem and progenitor cells serve as the cells of origin for breast cancer, understanding how these populations change with age is becoming a central focus in efforts to elucidate the link between aging and increased breast cancer risk (Table 2). In particular, age-related disruptions in stem and progenitor differentiation dynamics and lineage integrity may alter epithelial composition in ways that predispose the tissue to malignant transformation.

**Table 2:** Aging phenotypes in human and rodent mammary epithelial cells

Age related changes in mammary epithelial cells	
**Mouse/Rat**	**Human**
**Basal/MaSCcompartment:** • Expansion of MaSC-enriched basal population (CD49f^hi^) [34, 91, 92] • Aged basal cells lose identity (↑ luminal markers, ↓ basal markers) [93–95] • Reduced reconstitution potential [92], downregulation of stem genes [96], activation of senescence [97] • Bcl11b loss → premature aging, basal expansion, increased senescence; enhanced DMBA tumor susceptibility reversed by NF-κB/Jak-Stat inhibition [97] • Accumulation of KRT6A/IL33 + hybrid cells in the basal layer [59]	**Basal/MaSCcompartment:** • Luminal cells acquire basal features (KRT5/14) with age [98] • Enrichment of KRT5/14-expressing luminal progenitors in older women [99] • ↑ basal adhesion, migration, MAPK activation [93] • Aged basal cells drive luminal transcriptional/methylation changes via GJB6-mediated communication [100]
**Luminal compartment:** • Aged luminal cells show ↑ basal gene expression, ↓ luminal signatures [91, 94] • Emergence of ‘aging-LPs’ with intermediate basal-LP state [94] • Midkine treatment of young rats phenocopies aged features [94]	**Luminal compartment:** • Accumulation of basal-luminal (BL) cells with age [99, 101] • BL cells express mixed lineage markers (LASP, LHS, basal) [99] • TGF-βRI signaling regulates BL phenotype [98]
**Epigenetic changes & Cancer risk:** • Bcl11b loss drives senescence and tumor susceptibility [97] • Senescent basal cells promote tumor formation [97] • IκB kinase (IKK) inhibitor TPCA-1 reverses aging and reduces tumor burden [97] • Expression of genes upregulated with age in mice is increased in human breast tumors (Luminal A and Luminal B) [91]	**Epigenetic changes & Cancer risk:** • Age-dependent DNA methylation loss at basal TF sites in luminal cells → basal marker expression, seen in luminal breast cancers [102] • ELF5 methylation clock reflects biological age and is accelerated in BRCA1/2 or PALB2 carriers [103] • BL gene signatures associated with basal-like tumors → potential cells of origin [98]

Aging has been consistently shown to coincide with an expansion of the MaSC-enriched basal population [34, 59, 91, 92]. These aging-specific CD49f^hi^ cells possess reduced in vivo reconstitution potential [92], decreased expression of stem-related genes [96], and distinct senescence programs [97] (Table 2). Of note, *Bcl11b*, a transcriptional repressor implicated in mammary stem cell maintenance [104, 105], was identified as a critical mediator of inducing age-associated senescence programs [97]. Loss of *Bcl11b* through a *Krt14-Cre; Bcl11bfl/fl* mouse model resulted in premature aging phenotypes in young mice, as indicated by increased senescence (e.g., elevated β-galactosidase activity and p16^Ink4a^ staining in MECs) and an expansion of the basal population. *Bcl11b* KO cells were also intrinsically more susceptible to DMBA-induced tumor formation, and this phenotype appeared to be driven by the accumulation of senescent cells. Inhibition of BCL11B’s downstream targets (NF-κB and JAK-STAT signaling) via the dual-function inhibitor TPCA-1 reversed aging phenotypes in old WT mice and young mice lacking *Bcl11b*, in addition to significantly reducing tumor burden in medroxyprogesterone acetate (MPA)- and DMBA-induced cancer initiation models [97]. In addition to a decline in functional capacity, aged basal cells also appear to display a loss of cellular identity, such as elevated expression of luminal lineage markers [106] and decreased expression of basal lineage markers [34, 91].

Evidence for an age-dependent loss of lineage integrity has also been identified within the luminal lineage [94, 95]. Through scRNA-seq analysis of rat mammary glands across aging, Yan et al*.* found that luminal cells display higher expression of basal signature genes and lower expression of luminal signature genes as they age [94]. Luminal cells from aged rats also displayed greater cell-to-cell distance compared to young, perhaps reflecting increased heterogeneity. Notably, the authors identified a distinct population of luminal progenitors (LPs) that emerge in aged glands. These LPs, termed aging LPs, occupied a pseudotime trajectory between the basal and main LP clusters, which may suggest that aging LPs represent a less-differentiated LP population. Young mice treated with Midkine (*Mdk*), one of the top differentially expressed genes upregulated in aged rat MECs, phenocopied aspects of aged mammary glands, such as an expanded basal population and accumulation of aging LPs [94].

The acquisition of basal characteristics in aged luminal cells has also been well documented in human tissues [91, 93, 95, 98, 99, 101]. Notably, an integrated single-cell transcriptomic and proteomic analysis of human breast tissue from 16 donors identified a population of alveolar luminal cells that accumulate with age and express both luminal and basal cell markers [98]. Specifically, these cells, termed basal-luminal (BL) cells, expressed traditional lineage markers from LASP (e.g., *CD14* and *FOLR1*), LHS (e.g., *MUC1, SERPINA1,* and *AGR2/3*), and basal (e.g., *KRT5/14/17* and *CAV1*) populations. A similar population of *KRT5/14*-expressing luminal progenitors with reduced lineage specific marker expression were shown to be enriched in human breast samples from older women [99]. The luminal progenitors from older women have also been shown to be more functionally similar to basal/myoepithelial cells, as demonstrated by their enhanced basal adhesion properties, migration, and MAPK signaling activation [93]. In addition to their mixed-lineage gene expression profile, BL cells have high expression of the progenitor marker *ALDH1A3,* and some were positive for the plasticity marker CD73, perhaps reflecting an immature cell state. Through cyclic immunofluorescent staining, the authors revealed further heterogeneity within the BL population. One BL population (BL1) differed from the other (BL2) by elevated expression of basal markers and decreased expression of alveolar luminal markers, potentially suggesting that BL cells occupy a spectrum of cell states among epithelial lineages. Of particular interest, the authors found that the unique gene signature of BL cells was significantly associated with basal-like breast tumors. Functional investigation in in vitro organoid cultures revealed the BL phenotype is directly regulated by TGFβRI signaling. Given the diverse role of TGFβ signaling in mammary gland development, cellular plasticity, and breast cancer, further investigation into the origins of BL cells, their age-dependent accumulation, potential connections to breast cancer, and dependence on TGF-β signaling is necessary.

These age-specific phenotypes of decreased cellular function and gain of mixed lineage markers have been suggested to recapitulate early stages of tumorigenesis [107] and/or reflect how aged cells are epigenetically primed for oncogenesis [100, 102, 103, 108, 109]. Consistent with this notion, DNA methylation profiling of young and aged luminal epithelial cells revealed consistent age-dependent changes in methylation patterns in transposable elements (TEs) at lineage-specific transcription factor binding sites [102]. Specifically, methylation loss at TE that are enriched in basal-lineage specific transcription factor binding sites promotes expression of basal markers in luminal cells and are often observed in luminal breast cancers, suggesting a directional relationship between aging and increased breast cancer susceptibility. While these observations are specific to the luminal lineage, basal cells have been implicated as key players in mediating the age-associated loss of lineage fidelity in luminal cells [100, 101]. Interestingly, basal-luminal co-culture experiments suggest that luminal cells adopt a transcriptomic and methylation profile that is dependent on the donor age of the basal cells with which they are in contact [101]. Further investigation into the molecular drivers of this age-specific cell–cell communication system identified an upregulation of the gap junction protein *GJB6* (*connexin-30*) in aged basal cells, and loss of which was sufficient to reduce the biological age of luminal cells in a basal-luminal co-culture system [100]. DNA methylation and expression of luminal lineage markers have also been proposed as useful predictors of biological age and breast cancer risk [103, 110–112]. *ELF5*, the luminal-specific transcription factor, decreases in expression and increases in methylation with age [103], making the gene an attractive candidate as a biomarker for age. Indeed, the *ELF5* molecular clock model proved superior to established pan-tissue DNA methylation models (Horvath clock and MEAT DNAm clock), providing a more reliable prediction of age in average-risk women (error = 2.9y versus 8.3y and 9.4y in the Horvath clock and MEAT DNAm clock, respectively). Of particular interest, when applied to high-risk samples that contain germline mutations in *BRCA1, BRCA2,* or *PALB2*, the ELF5 clock often predicted biological ages higher than chronological age, suggesting that significant discrepancies between biological and chronological age could serve as an indicator of breast cancer risk.

### Stem and progenitors in the basal lineage

The basal lineage is composed of multiple sub-populations with distinct molecular features and functional properties (see Table 1). While the basal and luminal populations in the adult mammary gland are predominantly maintained by lineage restricted progenitors, recent lineage tracing studies argue for the existence of long-lived MaSCs with bipotent potential. For example, 3D immunofluorescence imaging of cleared mammary glands demonstrated that long-lived MaSCs, such as those expressing *Bcl11b, Procr,* or *Tspan8*, possess bipotent abilities in the post-natal mammary gland [11, 41, 45, 76]. PROCR + basal cells differ from their PROCR- counterparts both functionally and phenotypically, with enhanced mammary gland reconstitution potential and considerable upregulation of mesenchymal-related genes (e.g., *Vim, Zeb1/2, Foxc2*) [45]. Despite their enrichment of epithelial-to-mesenchymal transition (EMT) transcriptional programs, PROCR + cells express markedly lower levels of basal keratins, such as *Krt14* and *Krt5*. Analysis of developing mammary glands from *Procr-CreERT2-IRES-tdTomato* mice revealed that *Procr* + cells are sparsely and intermittently distributed along the mid-to-distal regions of embryonic mammary ducts, as well as throughout mature ducts in both pre- and post-pubertal glands. Despite their absence in TEBs, which are composed of highly proliferative progenitors, *Procr* + MaSCs are largely proliferative, as demonstrated by their robust EdU incorporation in 8-week old mammary gland ducts following a 3-h chase. In addition, a recent study demonstrated that *PROCR* + cells are enriched in expression of the Programmed death ligand (PD-L1) [44]. PD-L1 + basal cells had high regenerative capacity in transplantation assays as compared to PD-L1- cells and lineage tracing studies demonstrated that *Pd-l1* + cells like *Procr* + cells have bipotent characteristics. This is an intriguing discovery given the role of PD-L1 in immune evasion [113] and the fact that *Procr* + marks a specific cancer stem cell population in triple-negative breast cancer [114].

Quiescent MaSCs represent a highly heterogeneous population despite their relatively small overall contribution to the total number of epithelial cells in the mammary gland. While *Procr*-expressing MaSCs represent a cycling population [45], *Bcl11b*-expressing MaSCs are largely quiescent under homeostatic conditions [104]. Compared to *Procr* + MaSCs, *Bcl11b* + MaSCs were significantly less proliferative, as demonstrated by their reduced percentage of cells in the 4 N DNA fraction, lack of EdU incorporation, and the high expression of *Bcl11b* in Pyronin Y^low^-Hoechst^low^ G_0_ MECs. In vivo transplantation assays using *Bcl11b* reporter mice showed that *Bcl11b*^*high*^ cells have higher reconstitution potential as compared to *Bcl11b*^*neg*^ cells [104]. Recent lineage tracing studies using *Bcl11b-rtTA/TetO-Cre/R26-tdTomato* mice have demonstrated that *Bcl11b* labels bipotent MaSCs in the embryonic and adult mammary gland and these cells persist after multiple rounds of pregnancy [41]. Moreover, *Bcl11b* expression is enriched in CD200 + CD34 − basal cells whereas the CD200-CD34 + basal cells represent a unipotent basal progenitor [41]. In addition, CD1d marks a minority population of quiescent MaSCs that make up just 1% of the CD24^+^CD29^h^ MaSC population and demonstrate robust reconstitution potential (MRU frequency of ~ 1/8 compared to 1/44 in Lin^−^CD24^+^CD29^h^ cells) [43]. Expression of *Bcl11b* was also found to be enriched (fivefold) in CD1d + MECs compared to CD1d- [104]. However, the functional contributions of CD1d + MaSCs to mammary gland development and homeostasis remains undetermined.

Similar to the *Bcl11b* + and CD1d + MaSCs, a subset of *Lgr5* + MaSCs that co-express *Tspan8* remain dormant in the adult mammary gland, but can become activated in response to hormones [11]. *Lgr5* +/TSPAN8 + cells have higher repopulating ability in transplantation assays (~ 1 in 16 for single sorted cells) as compared to *Lgr5* +/TSPAN8- cells (1 in 63) and are largely quiescent by Pyronin Y/7-AAD staining. The authors also used EdU label-retention to trace the link between embryonic mammary cells and adult MaSC subsets. EdU was administered to *Lgr5-GFP-IRES-creERT2* mice between E14.5-E18.5, and analysis six weeks later showed that label-retaining cells were mainly in the *Lgr5* + TSPAN8^hi^ population. These cells stayed in the proximal gland region 11 weeks after labeling indicating that embryonic label-retaining cells are quiescent in the adult mammary gland. Interestingly, the authors demonstrate that EdU labeling just before pregnancy leads to a 20-fold rise in cycling *Lgr5* + TSPAN8^hi^ cells, suggesting that the normally dormant Lgr5⁺Tspan8^hi^ population is highly hormone-responsive. While *Lgr5* +/TSPAN8 + and *Bcl11b* + MaSCs appear to be spatially enriched at nipple-proximal regions, it is unclear whether these populations overlap or are distinct from one another.

Downstream of MaSCs in the mammary epithelial differentiation hierarchy are equally diverse sub-populations of basal-restricted progenitors. *Sema3a* + basal progenitor cells (CD200-CD34 +), which are often localized to TEBs, display long-term contribution exclusively to the basal lineage, even after multiple rounds of pregnancy [41]. By leveraging the high mitochondrial activity intrinsic to highly clonogenic basal cells and a droplet-based proteomic analysis pipeline, CD36 was identified as a marker of basal progenitors with increased fatty acid uptake [48], potentially reflecting the distinct metabolic signatures identified in stem and progenitor populations [15, 115–117]. However, in situ identification and functional investigation of CD36 + basal progenitors in the mammary gland has yet to be established.

Multiple lineage tracing studies using basal lineage markers have also suggested that the basal lineage is overwhelmingly maintained by lineage-restricted progenitors in the postnatal mammary gland [52, 118]. Genetic lineage tracing using either the *Krt5* or *Krt14* basal lineage promoters revealed exclusive labelling of basal cells when pulsed during puberty and adulthood, except a minority population of luminal cells when mice were pulsed at P1 and analyzed following a 5-week chase [52]. Similar results were obtained using an alternative basal-specific lineage marker, *Acta2* [118]*.* These data strongly argue against the existence of bipotent stem cells in the basal lineage, and that the lineage restriction within the basal population occurs near the time of birth.

Similar studies using identical lineage-specific promoters have produced observations in direct conflict to those mentioned previously [76]. Hence, alternative lineage tracing strategies (see Table 3) that do not rely on previous assumptions about the specificity and consistency of lineage marker expression have provided a more unbiased approach to address the outstanding question of whether bipotent stem cells exist in the postnatal mammary gland. Utilizing *R26*^*[CA]30SYNbglA*^ and *R26*^*[CA]30EYFP*^ mouse models to randomly label a single MEC and trace its progeny through mammary gland development, Davis and Lloyd-Lewis et al*.* found that all clone clusters identified were of basal or luminal lineages but never both, again supporting the hypothesis that the basal and luminal lineages are independently maintained [122]. However, it is important to note that this labeling method inherently relies on cell division, rendering it incapable of tracking quiescent populations. Thus, the authors acknowledge that it remains possible that a quiescent population of bipotent stem cells exist.

**Table 3:** Methods to assay mammary stem cells

Methods to assay mammary stem cells	Advantages	Disadvantages/Limitations
**Transplantation Assay** [37, 38, 119]	• Functional assay for regenerative potential • No complex genetic models required • Relatively easy to implement • Can be applied to human breast epithelial cells	• Non-physiological context • Loss of 3D architecture can alter cell state and induce injury response • Cannot be used to map steady state kinetics at different developmental stages
**Cre-loxP Genetic Lineage Tracing (Constitutive Cre)**	• Physiological • Permanent heritable labeling • High specificity if promoter is well chosen	• No temporal control • Promoter activity may be broader/nonspecific • Labelling efficiency for knock-in lines is dependent on the promoter driving expression of Cre • Reporter activation can be incomplete or leaky, confounding results
**Inducible Cre-loxP—CreER, CreERT2, rtTA models** [52, 55, 75, 76, 120]	• Temporal control of labeling that can be used to map different stages of mammary gland development • Pulse–chase experiments possible • All advantages listed for constitutive Cre	•Sparse labeling required to map cells at the single cell level •Tamoxifen dosage effects—variable recombination efficiency and high doses inhibit proliferation of mammary epithelial cells
**Multicolor Cre Reporters (e.g. Confetti)** [58, 67, 76, 121]	• Clonal resolution within tissue • All advantages listed for inducible Cre	• Recombination biased towards certain colors • Imaging-intensive • Can only track fate of proliferating cells
**Neutral, multicolor labeling, mutation induced clonal mark** [58, 121, 122]	• Unbiased labelling of cells • Clonal resolution within the tissue	• Can mainly be used to track fate of proliferating cells
**DNA barcoding using lentivirus** [12]	• No transgenics required • Local injection = spatial restriction • High-throughput clonal tracking • Quantitative contributions • Compatible with single-cell sequencing	• Variable transduction efficiency – may label one population preferentially over another • Loses spatial information during sequencing • Sequencing intensive

Additional studies using *R26-CreERT2* or lineage-specific promoter driven CreERT2 expression confetti mouse models to investigate lineage specification and potency have produced strong evidence against the existence of bipotent MaSCs in the postnatal mammary gland [58, 67, 121]. Unbiased/neutral lineage tracing (*R26-CreERT2; R26-Confetti* mouse model) at clonal/ultra-low density (< 1% of cells labelled) in mice pulsed at 3-weeks-old and analyzed at 5- or 8-weeks-old demonstrated that MaSC-derived clones were either of basal or luminal identity, but not both [58]. These results were also reproduced at higher doses of tamoxifen administration (termed “low-density” labeling) under a similar pulse/chase strategy (labelled at pre/early puberty → analyzed at late puberty/early adulthood) and during alveologenesis (labelled at 12wk pre-pregnancy → analyzed at lactation day 4–5) [121].

Wuidart et al*.* (2016) devised a rigorous statistical framework based on the *Krt14-CreERT2/R26-Confetti* mouse model to determine the probability that a unicolor pair consisting of a basal and luminal cell results from a bipotent MaSC or independent labeling of two neighboring cells by chance [67]. This model, which relies on (1) the probability of observing any unicolor pair and (2) the probability that any given labelled basal cell neighbors an independently labelled luminal cell, failed to produce any data supporting the existence of bipotent MaSCs in postnatal mammary gland development. To rule out the possibility that a minority population of bipotent MaSCs escape labeling at clonal or mosaic density, Wuidart et al*.* performed lineage tracing at saturation in *Krt14-rtTA/TetO-Cre/Rosa-YFP* mice. Across adult, pregnant, and lactating developmental stages, YFP + cells were nearly exclusive to the basal lineage (98.3–99.85%). Moreover, the small percentage of YFP + labeled luminal cells was stable from the initial 3 days of DOX treatment throughout all developmental time points analyzed (roughly 0.2% of luminal cells), suggesting that basal cells do not give rise to luminal cells. Likewise, lineage tracing of luminal cells using a *Krt8-rtTA/TetO-Cre/Rosa-tdTomato* mouse model at saturation demonstrated that the luminal lineage is maintained by long-term luminal stem cells, again arguing against bipotent MaSCs.

### Progenitors in the luminal lineage

The luminal epithelial lineage can be stratified into two major sub-lineages based on the presence or absence of hormone receptors and functional properties: Luminal Adaptive Secretory Precursor (LASP) (also referred to as alveolar/AV, secretory, milk-producing, or hormone-receptor low) and luminal hormone-sensing (LHS, also referred to as mature luminal or hormone-receptor high) (Table 1). The two luminal sub-lineages serve distinct functions in the mammary gland. As suggested by their name, LHS cells express ER/PR and play a critical role in receiving and transmitting the complex hormone signaling programs that orchestrate major stages of mammary gland development. LASP cells receive indirect hormone signals from neighboring LHS cells (e.g., AREG, WNT4, RANKL) and are primarily responsible for the expansion of alveoli and production of breast milk during reproductive stages. Just as they possess distinct functions, genetic lineage tracing experiments using LASP- and LHS-specific lineage markers [46, 68] and mathematical models of cell division kinetics [63] suggest that they are maintained through distinct pools of unipotent progenitors.

The LASP lineage is enriched for progenitors with high clonogenic capacity in vitro [31, 51, 123, 124]. Given this reputation of enhanced progenitor activity compared to most LHS cells, some scRNA-seq datasets have classified LASP clusters as luminal progenitors [16, 94, 125]. Nevertheless, this incomplete grouping fails to reflect the extensive heterogeneity contained by the LASP sublineage. Within the LASP progenitor population, there have been a number of genes reported to be uniquely expressed and can therefore serve as markers. Historically, these populations have been identified, isolated, and characterized by the cell surface markers of CD14, cKIT, CD49b, and CD61 [49–51]. However, the imperfect overlap of these markers underscores the complexity and diversity of luminal progenitors. For e.g. *Notch3* is expressed in the luminal lineage across all compartments but within the CD49b + population, *Notch3* expression enriches for cells with high clonogenic potential [55]. The transcription factor, *Elf5*, has also been documented to mark LASP or CD14 + hormone-receptor- (HR) low progenitors [126]. Through intravital imaging of pubertal *Elf5-rtTA/TetO-Cre/Confetti* mice, Dawson et al*.* tracked the spatial and temporal dynamics of *Elf5*-expressing LASP progenitors in TEBs and ducts [126]. They found that the LHS (HR +) progenitors had higher motility and went from a loose structure in TEBs into the rigid layer of conoid cells within ducts. In contrast, LASP (HR-) progenitors possess columnar morphologies and reduced motility in both TEB and ductal contexts, potentially implying a passive role during ductal elongation [126].

Compared to the LASP lineage, the LHS sublineage contains fewer progenitors, but they display high rates of proliferation to rapidly give rise to differentiated HR + luminal cells [69, 126]. While the independence of the LASP sublineage has been demonstrated through lineage tracing studies using *Sox9, Elf5, Wap,* and *Notch1* promoter driven *Cre* expression, the independent maintenance of the LHS sublineage has been established using *Prom1, Pgr,* and *Esr1* promoters [46, 68, 69, 126]. These LHS progenitors have also been identified and isolated through FACS by combining cell surface markers CD61/CD14/cKit with Sca-1/Ly6A [50, 51, 54] or CD133/PROM1 [34, 54, 126]. Using CD14 and Sca-1 to resolve LHS LPs (CD14 +/Sca-1 +), Dawson et al*.* found that this population makes up roughly 4% of all luminal cells in the adult mammary gland, underscoring their rarity [126]. Intravital imaging of TEB structures revealed that LHS (HR +) progenitors are highly motile and display elongated morphologies, but their differentiated progeny (HR + mature luminal/LHS cells) lack these functional characteristics [126]. These findings exemplify the functional differences among luminal sublineages and across the differentiation hierarchy. Future work must include thorough investigation of progenitor cell dynamics across space and time by leveraging innovative technologies such as intravital imaging coupled with genetic lineage tracing strategies.

### Conclusion and future perspectives

The past two decades have seen unprecedented advances in understanding mammary epithelial hierarchy and mammary stem cells since their first identification through transplantation assays nearly 60 years ago [127, 128]. These discoveries have been made possible through immense technological advances in genetic engineering of complex mouse models that allow temporal resolution during development, microscopy, flow cytometry and single-cell RNA-sequencing. Using different tools to identify stem cells and their dynamics through development have produced conflicting views on whether the mammary stem cells exist as multipotent cells beyond the early embryonic stages. However, the amalgam of these studies has also provided important insights into how stem cells behave in situ during homeostatic conditions versus under regenerative conditions. These differences have had significant implications in understanding cellular identity, multipotency, and transcriptional programs during injury repair [129] and breast tumor initiation/progression [4, 130–134].

While much of the work on understanding stem cell identity has focused on the epithelial compartment, the relationship to the niche remains an area of active investigation. The role of the extracellular matrix in specifying cell fate and malignant progression is well established [135–142], but its role in regulating stem and progenitor cells is only beginning to be understood [143, 144]. Recent studies have also shown fibroblast populations are dynamically remodeled throughout mammary gland development and have active roles during branching morphogenesis [19, 145]. A recently published study has for the first time spatially mapped the heterogeneity in fibroblasts populations during development and identified peri-TEB fibroblasts that are recruited by the growing epithelium from preadipocytes in the fat pad [146]. Moreover, recent studies have shown that both stromal and ductal macrophages actively cross-talk with stem cells in the basal lineage and are required to maintain the stem cell niche [42, 147].

Most studies examining mammary epithelial cells within their niche have concentrated on mammary glands prior to aging. In the mammary gland the niche undergoes extensive remodeling with pregnancy, lactation and involution that can further impact the identity and transcriptional programs of progenitor cells [82, 83, 148–150]. Moreover, recent studies have shown that the immune cells as well as the stromal microenvironment undergo significant changes with aging leading to an immune suppressive and senescent phenotype that can drive tumor progression [91, 151]. Given that breast cancer rates increase with age and an early pregnancy reduces long-term risk, understanding the epithelial cell dynamics in relation to aged niche is an area of active investigation [59, 91, 152]. The advent of spatial transcriptomics will allow us to understand the reciprocal regulation of stem cells and the niche as it evolves with pregnancy and age [153]. This combined with CRISPR based lineage tracing tools [154–156] that do not require promoter-based methods will enable us to generate high-resolution maps of the developmental hierarchy of mammary epithelial cells and their niches.

## Data Availability

No datasets were generated or analyzed during the current study.
